# Genome-Wide Identification and Characterization of the Laccase Gene Family in *Fragaria vesca* and Its Potential Roles in Response to Salt and Drought Stresses

**DOI:** 10.3390/plants13233366

**Published:** 2024-11-29

**Authors:** Jingjing Kong, Rui Xiong, Keli Qiu, Xinle Lin, Debao Li, Lijuan Lu, Junyong Zhou, Shufang Zhu, Mao Liu, Qibao Sun

**Affiliations:** 1 Key Laboratory of Horticultural Crop Germplasm Innovation and Utilization (Co-Construction by Ministry and Province), Institute of Horticultural, Anhui Academy of Agricultural Sciences, Hefei 230031, China; kjj0563@163.com (J.K.); m15255173358@163.com (R.X.); 18133495827@163.com (D.L.); llj229@163.com (L.L.); coplmn@163.com (J.Z.); sfchu319@126.com (S.Z.); ahyys@126.com (M.L.); 2 Anhui Provincial Key Laboratory for Germplasm Resources Creation and High-Efficiency Cultivation of Horticultural Crops, Hefei 230001, China; 3 National Engineering Laboratory of Crop Stress Resistance Breeding, Anhui Agricultural University, Hefei 230036, China; m1996qkl@163.com; 4 Anhui Province Key Laboratory of Horticultural Crop Quality Biology, Anhui Agricultural University, Hefei 230036, China; 13733989373@163.com

**Keywords:** *Fragaria vesca*, laccase, phylogenetic analysis, expression patterns, salt and drought stresses

## Abstract

Laccase (*LAC*, EC 1.10.3.2) is integral to the formation of lignin synthesis, flavonoid production, and responses to both biotic and abiotic stresses. While recent studies have characterized numerous *LAC* gene families and their functions across various plants, information regarding *LAC* genes in woodland strawberry (*Fragaria vesca*) remains limited. In this study, we identified a total of 57 *FvLAC* genes in the *Fragaria vesca* genome, which were phylogenetically categorized into five distinct groups. Analysis of the gene structures revealed a uniformity in the exon–intron structure among the subgroups, while conserved motifs identified unique motifs specific to certain subgroups, suggesting functional variations. Chromosomal localization studies indicated that *FvLACs* are distributed across seven chromosomes, and collinearity analysis demonstrated that *FvLACs* exhibit collinearity within the species. Additionally, cis-acting element analysis suggested that *FvLAC* genes are involved in stress responses, hormone responses, light responses, and the growth and development of plants. qRT-PCR demonstrated that *FvLACs* responded to salt, drought, and hormone stresses, with the expression levels of *FvLAC24*, *FvLAC32*, and *FvLAC51* continuously increasing under these stress conditions. Furthermore, transgenic yeast experiments revealed that *FvLAC51* enhanced yeast tolerance to both salt and drought stresses, while *FvLAC24* and *FvLAC32* negatively regulated yeast tolerance under these same conditions. These findings provide a theoretical foundation for further investigation into the functions of *FvLAC* genes in woodland strawberry.

## 1. Introduction

Laccase (*LAC*, EC 1.10.3.2) is a member of the multicopper oxidase family and contains a copper oxidase domain, which catalyzes the degradation of various aromatic or non-aromatic compounds (phenols, monolignols, lignins, anilines, thiols, and arylamines) [1]. In its catalytic mechanism, copper ions absorb electrons and reduce oxygen to water during the electron transfer process [2]. Laccases have been identified in bacteria, fungi, insects, and plants. Fungal laccases are capable of oxidizing and degrading phenolic lignin structural units and are involved in the recycling and utilization of natural lignin, making them widely used in the manufacturing industry [3]. In contrast, plant laccases primarily participate in defense-related processes, such as lignin synthesis in the cell wall and the polymerization of phenolic compounds; they also play crucial roles in plant growth and in conferring resistance to various stresses [4,5,6].

To date, laccase genes have been identified in various species, including *Arabidopsis thaliana* [7], citrus [8], pear [9], cotton [10], poplar [11], and rice [12]. Previous studies indicated that most plant laccases play a role in the oxidation of monolignols, which contributes to the formation of higher-order lignin structures. In *Arabidopsis*, *AtLAC4* and *AtLAC17* are involved in the constitutive lignification of floral stems. The silencing of *AtLAC17* specifically affects the deposition of G lignin units in fibers, while the unit specificity of *AtLAC4* remains less clear [7]. *AtLAC5* is essential for the biosynthesis of seed-coat protective neolignans through its cooperation with the AtDP1 protein [13]. The absence of *LAC8* and *LAC5* double mutants results in a significant decrease in lignin content and a notable increase in saccharification yield without impacting plant integrity in *Brachypodium distachyon* [14]. In *Populus deltoides*, laccase (*PdLAC2*) is associated with the oxidation of phenolics and the conjugation of flavonoids that interact with lignin in the cell wall [15]. In cotton, the *GhLAC15* gene enhances resistance to *Verticillium wilt* by increasing cell wall lignin content [16].

Laccases also play significant roles under abiotic stress conditions, including drought, salinity, heavy metal stress, and low temperature. In rice, the laccase *OsLAC10* enhances the tolerance of *Arabidopsis* to copper (Cu) stress, potentially through lignification in the roots, which prevents excessive copper absorption [12]. Additionally, a putative laccase precursor gene, *OsChI1*, has been shown to increase the tolerance of transgenic *Arabidopsis* to drought and salinity stress [17]. Recently, the overexpression of *PeLAC10* was demonstrated to elevate lignin content in transgenic *Arabidopsis*, thereby improving their adaptability to phenolic acid and drought stress [18]. Furthermore, *PeuLAC2* enhances drought tolerance in *Populus euphratica* by improving water transport capacity [5]. In *Poncirus trifoliata*, silencing *CsLAC18* significantly reduced the plant’s resistance to cold stress [19]. Overall, these studies indicate that laccases play a vital role in plant development and their responses to stress.

Strawberries are among the most popular fruits worldwide, known for their excellent taste and high content of sugars, vitamins, amino acids, and other essential nutrients [20]. Additionally, strawberry plants possess a large leaf area and shallow root distribution, and they are well recognized for their sensitivity to abiotic stresses [21]. The diploid woodland strawberry (*Fragaria vesca*, 2n = 2x = 14) has a relatively small genome (240 Mb) and a short growth cycle, which facilitates cultivation and reproduction. Furthermore, this species is increasingly regarded as an emerging model organism for research focused on the functions of specific genes and genomic analyses within the *Rosaceae* family [20]. In this study, we identified 57 members of the *LAC* gene family in woodland strawberry (*Fragaria vesca*). By analyzing the physical and chemical properties (position, protein length, molecular weight, isoelectric point, aliphatic index, grand average of hydropathicity, and subcellular localization) of these genes, as well as their protein structures and phylogenetic relationships, we clarified the evolutionary patterns of the *FvLAC* family members. Additionally, the analysis of the promoter sequence of *FvLACs* revealed the presence of 32 regulatory elements associated with stress responses, hormone responses, light response, and MYB-related and development-related processes. The treatments of 20 *FvLACs* in groups I to III with salt, drought, ABA, and MeJA demonstrated that all 20 genes exhibited varying degrees of response to these stressors. Experiments conducted on transgenic yeast indicated that the *FvLAC51* gene enhances tolerance to salt and drought stresses, whereas *FvLAC24* and *FvLAC32* appear to reduce tolerance under similar stress conditions. Subcellular localization studies confirmed that FvLAC24, FvLAC32, and FvLAC51 are situated in the cell membrane, classifying them as membrane proteins. These findings enhance our understanding of *FvLACs*’ functions and aid in the identification of candidate genes for further exploration of salt and drought stress tolerance in strawberry plants.

## 2. Results

### 2.1. Identification of Laccase Genes from Fragaria vesca Genomes

A total of 57 members of the *FvLAC* gene family were identified in *Fragaria vesca* by using HMMER3.0 (http://hmmer.org/download.html, accessed on 3 July 2024) and SMART (http://smart.embl-heidelberg.de/, accessed on 25 May 2024), and these were designated from *FvLAC1* to *FvLAC57* based on their chromosomal positions. Detailed information of the *FvLACs*, including gene name, gene ID, position, protein length, molecular weight (MW), isoelectric point (pI), aliphatic index, grand average of hydropathicity (GRAVY), and subcellular localization is shown in Table 1. Among the 57 *FvLAC* proteins, their length ranged from 440 (*FvLAC36*) to 616 (*FvLAC21*) amino acids, with the MWs varying from 49.20 kDa (*FvLAC36*) to 69.3 kDa (*FvLAC21*). The isoelectric point (pI) varied from 4.52 (*FvLAC44*) to 9.78 (*FvLAC40*), with 54% classified as basic proteins and 46% as acidic proteins. Furthermore, the aliphatic index of the *LAC* family proteins ranged from 72.29 (*FvLAC14*) to 94.5 (*FvLAC24*), and the GRAVY values ranged from −0.369 (*FvLAC29*) to 0.05 (*FvLAC38*), indicating that 96.5% of the *FvLAC* gene members are hydrophilic proteins (the value of GRAVY < 0). Predicted subcellular localization results indicated that all *FvLAC* family proteins were located in the cell membrane. Table 1 provides detailed information about the physiochemical proprieties of each identified *FvLAC* protein.

### 2.2. Phylogenetic, Gene Structure, and Conservation Motif Analysis of LAC Genes Among Fragaria vesca and Arabidopsis

**Figure 1:**
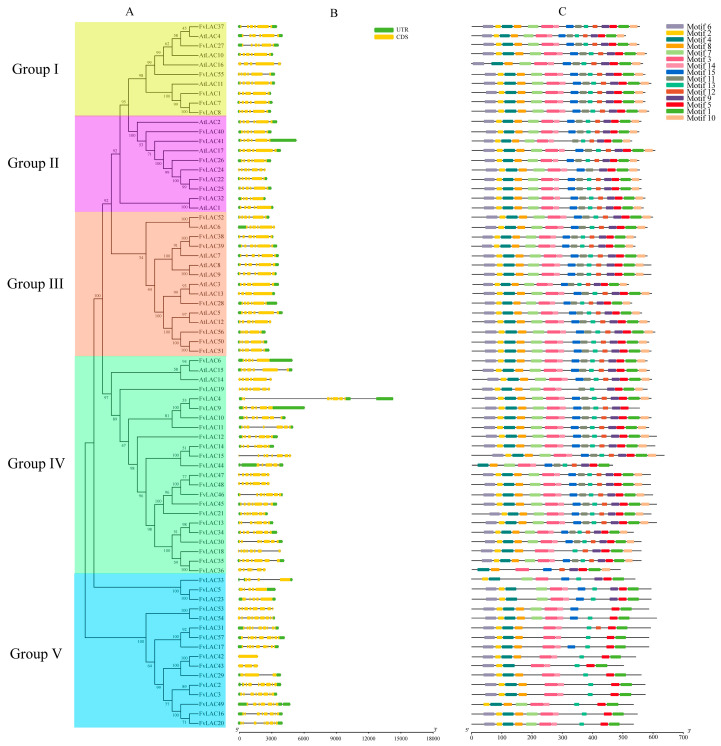
Phylogenetic relationships, gene structures, and conserved protein motifs of the *LAC* genes from *Arabidopsis* and *F. vesca*. (**A**) Phylogenetic tree of the *LAC* family. Various highlighted colors represent the different groups. (**B**) Exon–intron organizations of the *LAC* genes. Green boxes represent UTRs, yellow boxes represent exons, and black lines represent introns. (**C**) Conserved motif analysis of *LACs* within each group. Different color boxes represent different motifs.

To gain insight into the phylogenetic relationships of the *LAC* gene families, a phylogenetic tree was constructed using the neighbor-joining (NJ) method among 17 *Arabidopsis LACs* and 57 *FvLACs*. Based on the analysis of *Arabidopsis* laccases, the laccases in *Fragaria vesca* were clustered into five distinct groups (Figure 1A), revealing an uneven distribution among these groups. Group Ⅰ included six *FvLACs* (*FvLAC1*, *7*, *8*, *27*, *37*, *55*) and four *AtLACs* (*AtLAC4*, *6*, *10*, *11*). In group Ⅱ, seven *FvLACs* (*FvLAC22*, *24*, *25*, *26*, *32*, *40*, *41*) were clustered alongside three *AtLACs* (*AtLAC1*, *2*, *17*). Group Ⅲ comprised eight *AtLAC* members (*AtLAC3*, *5*, *6*, *7*, *8*, *9*, *12*, *13*) and seven *FvLACs* (*FvLAC28*, *38*, *39*, *50*, *51*, *52*, *56*). Group Ⅳ contained the largest number of *LACs*, consisting of 21 *FvLACs* (*FvLAC4*, *6*, *9*, *10*, *11*, *12*, *13*, *14*, *15*, *18*, *19*, *21*, *30*, *34*, *35*, *36*, *44*, *45*, *46*, *47*, *48*) and two *AtLACs* (*AtLAC14*, *15*). In contrast, group Ⅴ encompassed 16 *FvLACs* (*FvLAC2*, *3*, *5*, *16*, *17*, *20*, *23*, *29*, *33*, *37*, *42*, *43*, *49*, *53*, *54*, *57*) genes, with no *AtLAC* genes clustered with this group (Figure 1A).

The web server GSDS (Gene Structure Display Server) was utilized to analyze the gene structures of the *FvLACs.* As shown in Figure 1B, 40 *FvLACs* possessed two UTR regions, while 5 *FvLAC* genes (*FvLAC2*, *4*, *33*, *44*, *49*) contained three UTRs. Additionally, two *FvLAC* genes (*FvLAC54*, *55*) included only the 3′UTR region, and ten *FvLAC* genes (*FvLAC15*, *18*, *19*, *24*, *36*, *42*, *43*, *47*, *48*, *53*) lacked any UTR regions. Except for *FvLAC42*, the remaining 56 genes were broken genes, containing between three and nine exons in an uneven distribution. Furthermore, phylogenetic analysis revealed a similar exon–intron distribution pattern among genes with close evolutionary relationships, indicating that the gene structure of *FvLACs* corresponds to their evolutionary relationship. The conserved motif of all *FvLAC* proteins were constructed using the MEME (Multiple Em for Motif Elicitation) program, and a total of 15 conserved motifs (motif 1 to motif 15) were identified (Figure 1C). Apart from *FvLAC9*, all other *FvLACs* contained motif 1, while motif 3, motif 4, and motif 5 were widely distributed throughout the entire *FvLAC* family. Furthermore, the *FvLAC* proteins within the same group exhibited similar motif compositions, whereas genes from different groups displayed distinct compositions. In addition to group Ⅴ, most genes in groups Ⅰ, Ⅱ, and Ⅲ shared the same motifs, indicating a high degree of conservation in *FvLAC* gene family. In summary, the similarities in gene structures and conserved motif compositions within the same group forcefully supported the phylogenetic analysis for the group classifications.

### 2.3. Chromosomal Location, Genome Synteny, and Duplication of the FvLAC Genes

**Figure 2:**
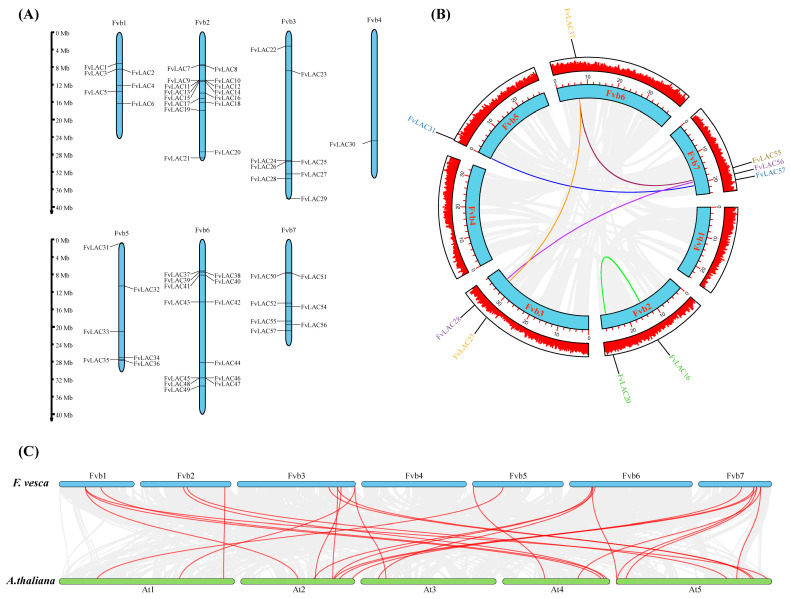
Chromosome distribution and collinearity analysis of *FvLACs.* (**A**) Chromosomal localization of 57 *FvLAC* genes. The chromosome numbers are indicated at the top of each chromosome image, and the scale on the left is in mega-bases. (**B**) Collinearity relationship analysis of the *FvLAC* gene family. The colored lines indicate duplicated *FvLAC* gene pairs. (**C**) Synteny analysis of *LACs* between *A. thaliana* and *F. vesca.* Gray lines in the background indicate all the collinear blocks, and red lines highlight the syntenic *LAC* gene pairs.

The chromosomal analysis revealed that 57 *FvLAC* genes were distributed across 1 to 7 chromosomes (Figure 2A). Among these genes, chromosomes 2 and 6 contained more than ten genes each, while six *FvLAC* genes were located on chromosomes 1 and 5. Eight *FvLACs* were distributed on chromosome 3, seven on chromosome 7, and chromosome 4 carried only one *FvLAC* gene. Additionally, some *FvLAC* gene members on chromosomes 1, 2, 3, and 6 were found to exist in the form of gene clusters (Figure 2A).

To elucidate the underlying mechanisms associated with the expansion of *FvLAC* genes in the woodland strawberry, a collinear analysis was conducted to investigate potential gene duplication patterns using BLAST and MCScanX methods. Five pairs of duplicate genes (*FvLAC16*/*FvLAC20*, *FvLAC27*/*FvLAC37*, *FvLAC28*/*FvLAC56*, *FvLAC31*/*FvLAC57*, and *FvLAC37*/*FvLAC55*) and one triple (*FvLAC27*/*FvLAC37*/*FvLAC55*) were found in the woodland strawberry genome, with the majority of these genes concentrated on chromosome 3. This distribution may be attributed to tandem replication or whole-genome duplication (WGD) (Figure 2B). To further investigate the evolutionary mechanisms of *FvLACs*, a comparative syntenic map of *F. vesca* associated with *A. thaliana* was constructed. As illustrated in Figure 2C, seventeen *FvLACs* showed syntenic relationships with those in *A. thaliana*, predominantly located on chromosomes 3 and 7. These findings reflected the intraspecific collinearity observed in woodland strawberry plants.

### 2.4. Promoter Cis-Elements of the FvLAC Gene Family

**Figure 3:**
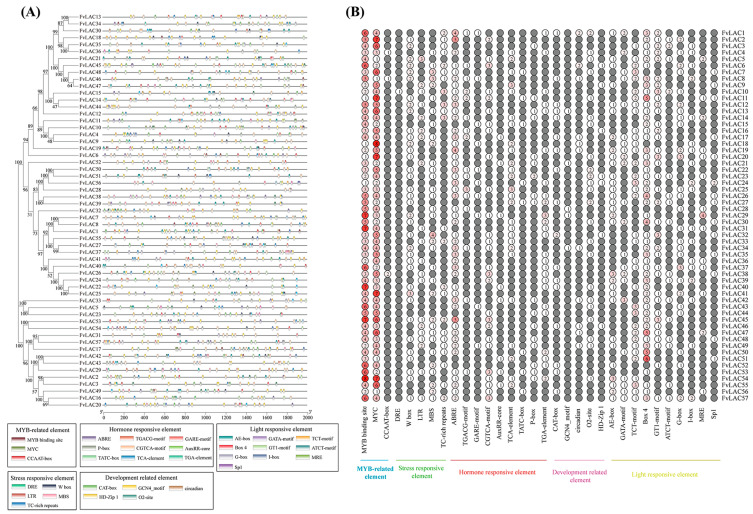
Identification of cis-acting regulatory elements in promoter region of *FvLACs*. (**A**) Cis-elements and their positions in the promoter region of the 57 *FvLAC* genes. Different colored boxes represent different cis-regulatory elements. (**B**) Number of each cis-acting element in the promoter region; the color intensity and numbers in the circles indicate the numbers of identified cis-acting elements.

The analysis of promoter cis-elements can provide insights into the regulatory functions and stress response modes of genes [22]. To gain a deeper understanding of the functions of *FvLAC* genes in the woodland strawberry, we predicted and summarized cis-acting elements based on the 2000 bp upstream promoter sequences. A total of 32 cis-regulatory elements were identified and categorized into 5 functional groups, including MYB-related, stress-responsive, hormone-responsive, development-related, and light-responsive elements (Figure 3A,B, Table S1). All *FvLAC* genes contained MYB-related, hormone-responsive, and light-responsive elements. The hormone-responsive cis elements identified included abscisic acid response elements (ABREs, 48 members), MeJA response elements (TGACG-motif and CGTCA-motif, 16 and 27 members, respectively), gibberellin-responsive elements (GARE-motif, P-box, and TATC-box, 10, 17, and 8 members, respectively), and auxin-responsive elements (AuxRR-core and TGA element, 8 and 25 members, respectively), which were present in nearly all *FvLAC* genes. Except for *FvLAC24*, *FvLAC43*, and *FvLAC44*, all *FvLACs* contained at least one sequence of stress-responsive cis-acting elements. Regarding development-related elements, we identified CAT-box elements (associated with meristem expression), GCN4-motif elements (associated with endosperm-specific expression), O2-site (associated with corn protein metabolism), circadian elements, and HD-Zip 1. Notably, *FvLAC2*, *FvLAC3*, *FvLAC41*, *FvLAC52*, and *FvLAC57* did not contain any of these five elements. Furthermore, the number of genes containing MYB/MYC binding site elements was the highest, followed by light-responsive (BOX 4) and abscisic acid response (ABRE) elements. Additionally, three genes (*FvLAC1*, *FvLAC45*, and *FvLAC57*) contained more than 30 elements, with *FvLAC45* having the largest number (32) of cis-elements, while *FvLAC36* and *FvLAC56* contained the fewest (15). All *FvLAC* genes, except *FvLAC44*, possessed elements from more than three categories. These findings suggest that *FvLAC* genes may play significant roles in various developmental processes and stress responses.

### 2.5. Candidate miRNAs Targeted to Regulate FvLAC Family Genes

MicroRNA (miRNA) is a small, non-coding RNA molecule consisting of 20–24 nucleotides. It plays a crucial role in regulating gene expression at the post-transcriptional level by binding to target genes and inhibiting their activity [23]. Previous studies have demonstrated that *LAC* genes can undergo miRNA-mediated post-transcriptional regulation in *Arabidopsis*, rice, and poplar [24,25,26,27]. To investigate the potential role of miRNAs in regulating *FvLAC* family members in the woodland strawberry, a total of 57 *FvLACs* were analyzed for the presence of potential miRNA target sites. As shown in Table 2, miRNAs were predicted for 16 *FvLAC* genes, and most of them were identified as miR397 and miR857. *FvLAC1*, *FvLAC32*, and *FvLAC55* might be regulated by miR397a only, while *FvLAC7*, *FvLAC8*, *FvLAC27*, *FvLAC28*, *FvLAC37*, *FvLAC38*, *FvLAC39*, and *FvLAC41* might be regulated by both miR397a and miR397b. In addition, miR875 might target *FvLAC3*, *FvLAC7*, *FvLAC23*, *FvLAC34*, *FvLAC41*, and *FvLAC52*, with only *FvLAC7* and *FvLAC41* potentially co-regulated by miR397a, miR397b, and miR857. These results suggest that miR397a/b and miR875 may play significant regulatory roles in the post-transcriptional regulation of *FvLACs*, contributing to the diverse functions and complex expression regulation of *FvLAC* genes.

### 2.6. FvLAC Expression Responds to Salt and Drought Stresses

**Figure 4:**
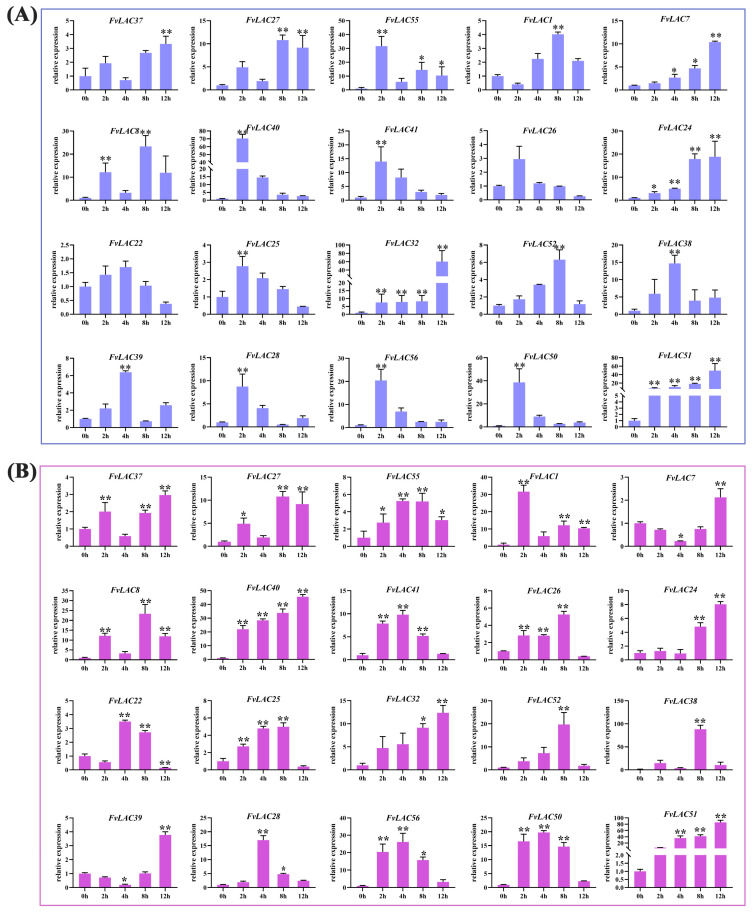
Expression analysis of 20 *FvLAC* genes under salt and drought treatments by qRT-PCR. (**A**) The 20 *FvLACs’* expression levels in seedlings under 300 mM NaCl treatments. (**B**) The 20 *FvLACs’* expression levels in seedlings under 20% PEG-6000 treatments (Sangon Biotech, Shanghai, China). Error bars indicate standard deviations among three independent biological replications. *FvActin* was used as the internal control, 0 h was used as the mock, and the *t*-test was used to analyze three biological replicates of each sample. *: *p*-value < 0.05, **: *p*-value < 0.01.

Salt and drought stresses are among the most prevalent abiotic stresses encountered by plants. To investigate the impact of these stresses on the *FvLAC* genes, we analyzed the relative expression of 20 *FvLAC* genes from groups I, II, and III in woodland strawberry subjected to salt and drought stresses using qRT-PCR. As illustrated in Figure 4A, under NaCl treatment, the expression levels of *FvLAC7*, *FvLAC24*, *FvLAC32*, and *FvLAC51* exhibited a continuous increase over a 12 h period, while the expression levels of the remaining 16 *FvLAC* genes displayed a wave-like pattern. For instance, *FvLAC40*, *FvLAC41*, *FvLAC26*, *FvLAC22*, *FvLAC25*, and *FvLAC52* demonstrated an initial increase followed by a decrease. Similarly, the expression trends of *FvLAC37*, *FvLAC38*, *FvLAC39*, *FvLAC29*, *FvLAC28*, and *FvLAC56*, along with the other 12 genes, were characterized by an initial upregulation followed by a decrease and a subsequent increase.

In alignment with the results from NaCl treatment, the expression levels of *FvLAC40*, *FvLAC24*, *FvLAC32,* and *FvLAC51* exhibited continuous increase after 12 h of PEG treatment. Additionally, seven *FvLAC* genes (*FvLAC55*, *FvLAC41*, *FvLAC25*, *FvLAC52*, *FvLAC28*, *FvLAC56*, *FvLAC50*) showed a trend of initial increase followed by a decrease, most with a peaking time at 4 h. *FvLAC7* and *FvLAC39* showed a trend of initial decrease followed by an increase, both reaching their lowest point at 4 h and their highest point at 12 h. The expression levels of the remaining seven *FvLACs* also displayed a wave pattern (Figure 4B). These results indicated that the 20 *FvLAC* genes respond differently to salt and drought stress over varying treatment times, which might contribute to their distinct biological functions under these stress conditions.

### 2.7. FvLACs Expression in Response to Hormone Treatments

**Figure 5:**
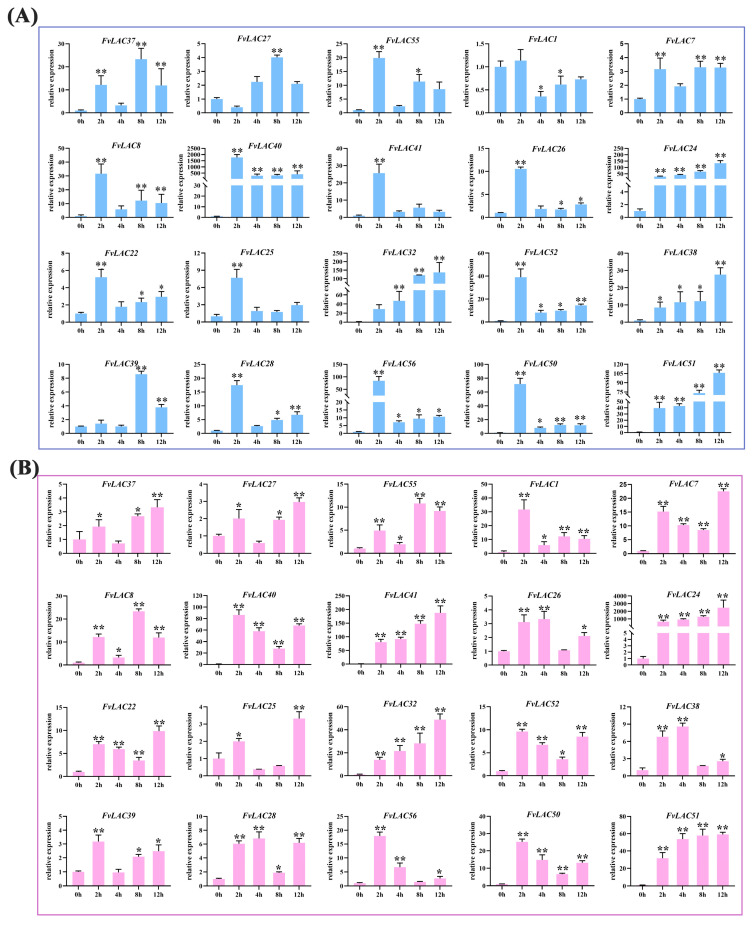
Expression analysis of 20 *FvLAC* genes under ABA and MeJA treatments by qRT-PCR. (**A**) The 20 *FvLACs’* expression levels in seedlings under 40 µmol/L ABA treatments. (**B**) The 20 *FvLACs’* expression levels in seedlings under 40 µmol/L MeJA treatments. Error bars indicate standard deviations among three independent biological replications. *FvActin* was used as the internal control, 0 h was used as the mock, and *t*-test was used to analyze three biological replicates of each sample. *: *p*-value < 0.05, **: *p*-value < 0.01.

The plant hormones abscisic acid (ABA) and methyl jasmonate (MeJA) play crucial roles in plant stress signaling pathways as well as in plant growth and development. To investigate whether the expression of *FvLAC* genes was induced by ABA and MeJA, we analyzed the expression patterns of these 20 genes in response to exogenous ABA and MeJA treatment using quantitative reverse transcription PCR (qRT-PCR). Under ABA treatment, the expression levels of 12 *FvLAC* genes peaked at 2 h before subsequently declining. These genes were *FvLAC1*, *FvLAC7*, *FvLAC8*, *FvLAC40*, *FvLAC41*, *FvLAC26*, *FvLAC22*, *FvLAC25*, *FvLAC52*, *FvLAC28*, *FvLAC56*, and *FvLAC50*. Notably, the expression levels of *FvLAC24*, *FvLAC32*, *FvLAC28*, and *FvLAC51* consistently increased over time with the treatment, while the expression of the remaining four *FvLAC* genes (*FvLAC37*, *FvLAC27*, *FvLAC55*, and *FvLAC39*) exhibited a fluctuating pattern (Figure 5A). Specifically, the expression levels of *FvLAC40* and *FvLAC24* increased more than 100-fold following ABA treatment, whereas the expression of the other genes increased by several- to dozens-fold.

Under 12 h MeJA treatment, in addition to the continuous increase in the expression of *FvLAC41*, *FvLAC24*, *FvLAC32*, and *FvLAC51*, the expression of the remaining 16 genes displayed 2 distinct fluctuating patterns. For example, *FvLAC37*, *FvLAC27*, *FvLAC7*, *FvLAC40*, *FvLAC26*, *FvLAC22*, *FvLAC25*, *FvLAC52*, *FvLAC38*, *FvLAC39*, *FvLAC28*, *FvLAC56*, and *FvLAC50* exhibited initial increases followed by decreases and subsequent increases (Figure 5B). Conversely, the expression of *FvLAC55*, *FvLAC1*, and *FvLAC8* increased initially, then decreased, and then increased and decreased again. Among these, the expression of *FvLAC24* increased by thousands of times, while the expression of the other genes increased by several- to tens-fold (Figure 5B).

### 2.8. The Tolerance of FvLACs to Salt and Drought Stresses in Yeast

**Figure 6:**
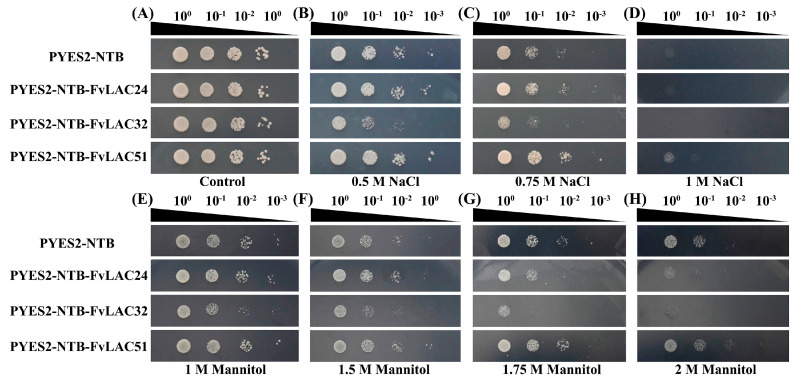
The function analysis of *FvLAC* genes under salt and drought stresses in the yeast strain INVSC1. (**A**) The growth of control and *FvLACs* yeasts under unstress condition. (**B**–**D**) The function analysis of control, *FvLAC24*, *FvLAC32,* and *FvLAC51* genes under different salt stresses in yeast strain INVSC1. (**E**–**H**) The function analysis of control, *FvLAC24*, *FvLAC32*, and *FvLAC51* genes under different drought stresses in yeast strain INVSC1.

Through our analysis of *FvLAC* gene expression under conditions of salt, drought, and ABA and MeJA treatments, we observed a continuous increase in the expression levels of *FvLAC24*, *FvLAC32,* and *FvLAC51* across all four treatments (Figure 4 and Figure 5). This observation prompted us to investigate the biological functions of these three genes further. The impact of *FvLAC24*, *FvLAC32,* and *FvLAC51* on yeast growth and stress resistance was investigated in yeast strains harboring the *FvLACs*-overexpressing construct (*PYES2*-*NTB*-*FvLAC24*, *PYES2*-*NTB*-*FvLAC32*, and *PYES2*-*NTB*-*FvLAC51*). The results indicated that both the control (pYES2-NTB) and the three overexpression yeast strains exhibited typical growth characteristics on SG-Ura medium, suggesting that the enhanced expression of *FvLACs* in INVSc1 strains did not adversely affect their growth under normal conditions (Figure 6A). However, in media containing 0.5 M, 0.75 M, and 1 M NaCl, the *FvLAC24* strain demonstrated a growth trend similar to that of the control, while the *FvLAC32* strain exhibited significantly reduced salt tolerance, showing almost no growth after a 1000-fold dilution at 0.5 M NaCl and a 100-fold dilution at 0.75 M NaCl (Figure 6B,C). In contrast, the *FvLAC51* strain displayed enhanced salt tolerance, while the other three strains perished without dilution under 1 M NaCl treatment. *FvLAC51* remained weakly resistant to NaCl, indicating its potential to enhance salt tolerance in yeast strains (Figure 6D).

Similarly, *FvLAC32* exhibited sensitivity to drought as mannitol concentration increased. In the presence of 1.75 M mannitol, the 10-fold diluted *FvLAC32* strain perished, whereas the control, *FvLAC24*, and *FvLAC51* strains continued to grow, with *FvLAC51* demonstrating the strongest growth potential and insensitivity to drought stress (Figure 6E–H). The *FvLAC24* gene exhibited higher sensitivity to both salt and drought stress in the presence of 1.75 M and 2 M mannitol compared with the control; these results suggest that *FvLAC24* and *FvLAC32* confer sensitivity to drought, while *FvLAC51* appears to enhance tolerance to this stress (Figure 6E–H). Overall, these findings indicated that *FvLAC32* may play a negative regulatory role under salt and drought stress, whereas *FvLAC51* may play a positive regulatory role.

### 2.9. Subcellular Localization of FvLAC Genes

**Figure 7:**
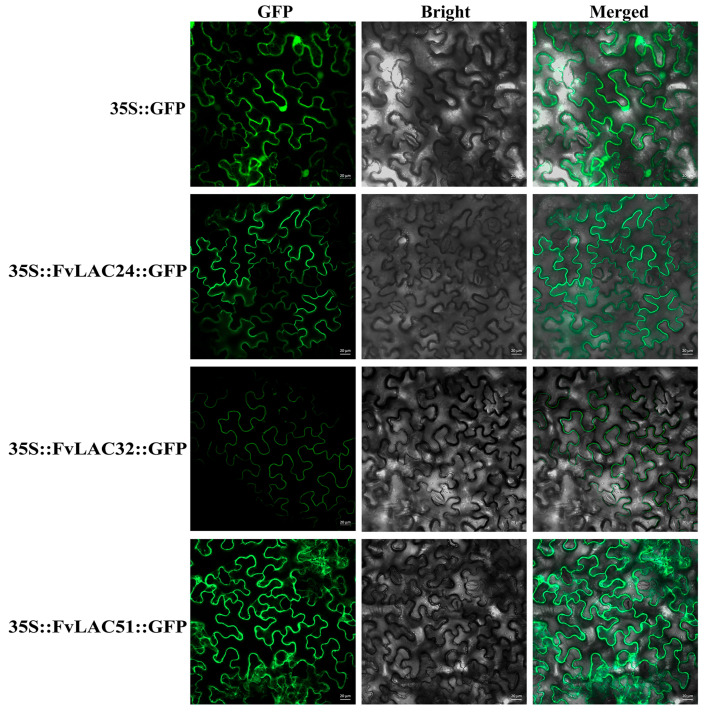
The subcellular localization analysis of FvLAC24, FvLAC32, and FvLAC51 proteins. GFP driven by the CaMV35S promoter was used as a control. Bars = 20 µm.

Subcellular localization provides crucial insights into a protein’s function. To further elucidate the function of FvLAC24, FvLAC32, and FvLAC51, we conducted a subcellular localization analysis. As illustrated in Figure 7, the green fluorescence of the empty vector (35S::GFP) was distributed on plasma membranes and in the nucleus, whereas the 35S::FvLAC24::GFP, 35S::FvLAC32::GFP, and 35S::FvLAC51::GFP fusion proteins were exclusively localized at the plasma membranes. This observation suggests that these three FvLAC proteins are likely localized at the plasma membranes. Furthermore, this result aligns with predictions made by the website and is consistent with findings regarding *LAC* protein subcellular localization in other species.

## 3. Discussion

Plant laccase is a multifunctional oxidase primarily involved in lignin synthesis, flavonoid metabolism, wound repair, plant growth, and response to adverse stress-related processes [28]. Since the laccase gene was first cloned from *Rhus vernicifera* in 1995, numerous plant laccase genes have been cloned and reported. To date, we have identified 17 *AtLACs* in *Arabidopsis* [29], 29 laccases in *Saccharum spontaneum* L. [30], 24 *CsLACs* in *Citrus sinensis* [8], 45 *BnLACs* in *Brassica napus* [31], 44 *GaLACs* in *Gossypium* spp. [10], 27 *SbLACs* in *Sorghum bicolor* (L.) [32], and 54 *EgrLACs* in *Eucalyptus grandis* [33]. However, the laccases present in strawberry plants remain unknown. In this study, we identified the laccase gene family in the woodland strawberry using bioinformatics methods, resulting in the identification of a total of 57 family members. According to the evolutionary tree, the gene structures, conserved motifs, and physicochemical properties of *FvLAC* proteins within the same group exhibited remarkable similarities. These observations align closely with the presence of *LACs* in various other species [8,9] (Figure 1A).

The exon–intron structures and conserved motifs exhibited similar regularities. The *FvLACs* in groups Ⅰ to Ⅳ had 5 to 7 exons, which aligns with the exon–intron structure observed in Arabidopsis and soybeans. This similarity suggests that the *FvLAC* gene structure in groups I to IV has remained highly conserved throughout evolution (Figure 1B). Notably, the genes in group Ⅴ were found to be distantly related to *Arabidopsis*, with exon numbers varying from 1 to 9, highlighting a small degree of diversity in *LAC* gene structure that may ultimately contribute to variations in gene function (Figure 1B). A previous study indicated that divergences in coding regions, particularly those capable of altering gene function, could result from substitutions or modifications of amino acids that affect exon–intron structures [34]. This would cause the *Arabidopsis* and strawberry *LAC* genes with different functions to cluster into different groups. It has been reported that *AtLAC4*, *AtLAC11*, and *AtLAC17* are necessary for lignin biosynthesis. Laccases of these two groups (*Arabidopsis* and strawberry) might be monolignol laccases that participate in lignin biosynthesis [7,35,36]. The genes encoding *AtLAC5*, *AtLAC12*, and *AtLAC13* were found to respond to ABA signals; *AtLAC6* was down-regulated when subjected to biological stress; and *AtLAC8* affected the plants’ flowering time [29], therefore, it was speculated that group Ⅲ *FvLAC* members may be related to plant flowering time, abiotic stress, and the ABA signaling pathway.

Tandem repetition and fragment duplication are the primary mechanisms involved in the replication of gene families, contributing to the emergence and functional diversification of novel genes [37]. Our results indicate that most *FvLAC* genes are concentrated on chromosomes 3 and 7, comprising seven clusters of tandem repeat genes across four chromosomes and five pairs of duplicate genes on five chromosomes. This distribution may be attributed to tandem replication or whole-genome duplication (Figure 2A,B).

While many studies have characterized plant laccase genes, the detailed regulatory mechanisms remain poorly understood. Cis-regulatory elements can be bound by transcription factors (TFs) to regulate the gene expression at the transcriptional level. Our analysis identified 32 types of promoters, including 3 MYB-related elements, 5 stress-related elements, 9 hormone-related elements, 5 development-related elements, and 10 light-responsive elements. Notably, all FvLAC promoters contain MYB-related elements, integral components of MYB transcription factors (TFs). Previous studies have shown that these MYB TFs play a crucial role in secondary cell wall formation, plant growth, and stress response [38,39,40]. In *Arabidopsis thaliana*, *AtMYB58/63* can activate the expression of *AtLAC4* by interacting with AC elements in the promoter, enhancing lignin synthesis and regulating secondary cell walls’ formation [41]. ABRE elements generally exist in the promoters of ABA hormone-responsive genes and can be bound by AREB/ABF or bZIP TFs [42]. W-box elements primarily exist in the promoter regions of resistance genes related to the resistance to disease, drought, low temperature, and salt, and they regulate plant resistance by mediating hormone signal transduction pathways [43,44]. These results suggest that *FvLACs* are regulated by a variety of factors and functions in numerous physiological processes, including development, morphogenesis, and responses to biotic and abiotic stress.

In plants, miRNAs are essential for growth, development, and stress responses. The levels of *LACs* can be controlled by miRNAs through post-transcriptional regulation. Previous research has indicated that Csi-miR397 functions as a suppressor of *LACs* in citrus [45]. Additionally, strawberry miR397 can modulate the expression of *LAC11a* during the fruit-ripening process [46]. The miR397-*LAC* module also plays a role in the domestication of rice [12]. We found that only 16 *LACs* among the 57 *LAC* members can be regulated by miRNAs, specifically miR397a, miR397b, and miR857 (Table 2). Notably, these miRNAs also target and regulate the *LAC* genes in citrus, suggesting that the miRNAs targeting the *FvLAC* gene family are relatively conserved. In summary, whether through transcriptional regulation, post-transcriptional regulation, or post-translational modification, the in-depth research on these regulatory factors and their mechanisms will enhance our understanding of the *FvLAC* gene regulatory network involved in plant development and stress resistance.

Plant laccases are known to be associated with cytokinin degradation and the flavonoid process, both of which are essential for plant growth [47,48,49]. Furthermore, many studies have demonstrated that laccase genes are responsive to various types of stress. In *Populus euphratica*, the overexpression of *PeuLAC2* c enhances drought tolerance by altering xylem structure and water transport [5]. Increased expression of the potential rice *LAC* family member *OsChl1* in *Arabidopsis* significantly improves plant tolerance to drought and salt stresses [17]. Our qRT-PCR analysis revealed that the expression of the 20 selected *FvLAC* genes was significantly induced by salt, drought, and ABA and MeJA treatments, indicating that *FvLACs* play a role in these stress responses and providing novel targets for enhancing the stress resistance in the strawberry. Notably, three *FvLAC* genes exhibited continuously elevated expression levels over 12 h under both salt and drought stresses, prompting us to conduct functional validation of these genes in transgenic yeast. As illustrated in Figure 6, *FvLAC24* and *FvLAC32* were found to weaken the salt and drought tolerance of transgenic yeast, whereas *FvLAC51* enhanced these tolerances (Figure 6A–H). These findings suggest that *FvLAC24* and *FvLAC32* may function as negative regulatory genes in response to salt and drought stress, while *FvLAC51* acts as a positive regulatory gene under similar conditions. In terms of evolutionary relationships, *FvLAC24* and *FvLAC32* are classified within group Ⅱ, while *FvLAC51* is categorized in group Ⅲ (Figure 1A); this clustering indicates that the functions of *FvLACs* within the same group are relatively conserved. Moreover, *FvLAC32* displayed significantly greater sensitivity to salt and drought compared with *FvLAC24*, which may be attributed to its higher expression levels following stress treatment (Figure 4A,B). In conclusion, the findings of this research provide a systematic understanding of the *FvLAC* gene family in the woodland strawberry and enhance our comprehension of the roles of *FvLAC* genes in relation to salt and drought stress. However, the specific biological functions of *FvLAC* in response to salt and drought stresses in the woodland strawberry remain unclear, necessitating further exploration in future research.

## 4. Materials and Methods

### 4.1. Identification of LAC Family Genes in the Woodland Strawberry Genome

The whole genome sequence, coding sequence (CDS), and protein sequence of *Fragaria vesca* (v4.0.a2) were downloaded from the GDR database (https://www.rosaceae.org/search/genes (accessed on 21 July 2023)). The 17 *AtLAC* protein sequences in *Arabidopsis* were obtained from the TAIR database (https://www.arabidopsis.org/ (accessed on 21 July 2023)). Hidden Markov model (HMM) profiles (PF007731, PF00394, PF07732) of the *LAC* family were downloaded from the InterPro database (https://www.ebi.ac.uk/interpro/ (accessed on 21 July 2023)), and a local protein database was constructed for searches using Hmmer 3.0 with a predefined threshold of E < 1 × 10^−3^. Candidate proteins and domain verification were ultimately confirmed through the SMART website (http://smart.embl-heidelberg.de/ (accessed on 22 July 2023)). The ExPASy ProtParam tool (https://web.expasy.org/protparam/ (accessed on 22 July 2023)) was utilized to calculate protein length (number of amino acids [aa]), molecular weight (MW), isoelectric point (pI), aliphatic index, and grand averages of hydropathicity (GRAVY). The CELLO v.2.5 website (http://cello.life.nctu.edu.tw/ (accessed on 22 July 2023)) and Cell-PLoc 2.0 (http://www.csbio.sjtu.edu.cn/bioinf/Cell-PLoc-2 (accessed on 22 July 2023)) were used to speculate the subcellular localization of proteins.

### 4.2. Phylogenetic Tree, Multiple Sequence Alignments, and Gene Structure Analysis

The phylogenetic tree was constructed based on sequence data using the neighbor-joining method with 100 bootstrap replicates via MEGA-X software (https://www.megasoftware.net/) [50]. The Gene Structure Display Server 2.0 (http://gsds.gao-lab.org/ (accessed on 5 August 2023)) was employed to obtain the exon–intron structures of the *FvLACs* [51]. Conserved motifs were analyzed by using the online tool MEME (https://meme-suite.org/meme/ (accessed on 5 August 2023)), and both the gene structure and conserved motif map were generated through TBtools analysis and visualized using Adobe Illustrator 2020 software [52,53].

### 4.3. Chromosome Distribution and Collinearity Analysis of FvLACs

The chromosomal location information of the *FvLAC* genes was acquired from the gff3 annotation file of the woodland strawberry genome and was graphically visualized using TBtools [53]. To investigate the evolution of *LAC* genes, gene duplication and collinearity relationships were identified via MCScanX, and intergenomic synteny analysis was conducted between *Arabidopsis thaliana* and *F*. *vesca* [54]. The syntenic diagram was also generated using TBtools.

### 4.4. Promoter Cis-Acting Elements and Prediction of miRNA Targets in FvLACs

The 2000 bps upstream of the gene-coding sequence was extracted as the promoter region of *FvLACs* by the Gtf/gff3 Sequences Extractor option in TBtools [53]. Cis-acting element analyses were predicted through PlantCARE online tool (https://bioinformatics.psb.ugent.be/webtools/plantcare/html/ (accessed on 7 August 2023)), and the common functional elements were visually displayed via TBtools [22,53]. The online database psRNATarget server (https://www.zhaolab.org/psRNATarget/ (accessed on 7 August 2023)) was used to predict miRNAs with target sites in *FvLAC* genes, selecting those with an expectation number under 5 [55].

### 4.5. Plant Materials, Growth Conditions, Abiotic Stress Treatments, and Tissue Collection in F. vesca

The woodland strawberry (Ruegen) seeds used in this study were provided by Zhao Mizhen from the Jiangsu Academy of Agricultural Sciences. After germination, strawberry seedlings were transplanted into pots containing a mixture of soil and vermiculite (1:4) and grown in a phytotron under conditions of 16 h light /8 h dark at 22 °C, with light intensity of 12,000 lux and a relative humidity of 65%. Following a uniform growth period of 10 weeks, the seedlings were used for drought stress, salt stress, and hormone treatments concurrently. Specifically, 20% PEG 6000 and 300 mM NaCl solutions were employed to irrigate the roots to simulate drought and salt stresses, respectively [56,57]. In addition, 40 µmol/L ABA and 40 µmol/L MeJA solutions were sprayed on leaves to induce hormonal stresses. Samples consisting of the whole plant were collected at designated time points (0 h, 2 h, 4 h, 8 h, or 12 h) following stress and hormone treatments, with 0 h serving as the control. Each sample comprised three biological replicates, with each replicate including six plants. The collected samples were promptly wrapped in clean tinfoil, rapidly frozen in liquid nitrogen, and stored at −80 °C until further analysis.

### 4.6. RNA Extraction and Quantitative Real-Time PCR (RT-qPCR) Analysis

Total RNA from all samples was extracted using an RNAprep Pure Plant Plus Kit (Tiangen Biotech, Beijing, China) according to the manufacturer’s protocols. The quality and purity of RNA were evaluated by 1% agarose gels and a Nanodrop One microvolume UV-Vis spectrophotometer (Thermo Scientific, Waltham, MA, USA), with a 260/280 ratio ranging from 1.8 to 2.1 and deemed acceptable for reverse transcription. Total RNA was reverse transcribed into cDNA utilizing the EasyScript^®^ One-Step gDNA Removal and cDNA Synthesis SuperMix Kit (Transgene, Beijing, China), and all cDNA samples were stored at −20 °C. Primers for candidate genes were designed using Primer Premier 5 software and NCBI-Primer Blast (https://www.ncbi.nlm.nih.gov/tools/primer-blast/ (accessed on 2 October 2023)), with FvActin and Fvtubulin serving as reference genes for RT-qPCR analysis (Table S1). The RT-qPCR reactions were conducted in a final volume of 15 μL f using PerfectStart^®^ Green qPCR SuperMix (Transgene, Beijing, China), and the reaction program was executed in the ABI7500 thermal cycler (Applied Biosystems, Foster City, CA, USA). Relative expression levels were calculated using the 2^−ΔΔ^CT method. The gene expression data are presented as mean ± SD and were analyzed for significant differences using ANOVA in GraphPad Prism 8 (NS: not significant; * *p* < 0.05; ** *p* < 0.01). Every experiment was conducted with three biological replicates, each of which was performed in triplicate.

### 4.7. Salt and Drought Stress Treatments of Transgenic Yeasts

The open reading frame (ORF) containing the termination codon of *FvLAC24*, *FvLAC32,* and *FvLAC51* was amplified by PCR from the cDNA of the ‘Ruegen’ strawberry and subsequently cloned into the *pYES2-NTB* vector to create the fusion plasmids *PYES2*-*NTB*-*FvLAC24*, *PYES2*-*NTB*-*FvLAC32*, and *PYES2*-*NTB*-*FvLAC51.* The empty vector, along with the three fusion vectors, was transformed into the yeast strain INVSC1, which was then plated on SD-Ura medium and cultured at 29 °C for 3–4 days. Positive clones were dissolved in SD liquid medium and cultured until the OD_600_ reached 1.2 to 1.4. Following centrifugation, the cells were resuspended in SD-Ura liquid medium devoid of a carbon source and subjected to shaking culture for 3 h. After a second centrifugation, the cells were resuspended in SG-Ura containing 2% galactose and incubated for 8 to12 h. The cells were then centrifuged again, and the OD_600_ of the yeast cell suspension was adjusted to 0.45. Serial dilutions (10^0^, 10^−1^, 10^−2^, 10^−3^) were performed, and the diluted samples were plated on SG-Ura agar plates containing varying concentrations of NaCl (0.5 M, 0.75 M, and 1 M) and mannitol (1 M, 1.5 M, 1.75 M, and 2 M) [58]. The plates were subsequently incubated at 29 °C for 3 days, and the growth of yeast colonies was observed and recorded [59].

### 4.8. Subcellar Localization Assays

The open reading frames (ORFs) of *FvLAC24*, *FvLAC32,* and *FvLAC51*, excluding the termination codon, were amplified via PCR from cDNA of ‘Ruegen’ strawberry and subsequently cloned into the pMDC43 vector to construct the fusion protein. The recombinant vectors, along with empty vector plasmids, were then transferred into *Agrobacterium tumefaciens* strain GV3101 and transiently expressed in tobacco (*Nicotiana benthamiana*) leaves. From 48 to 72 h after injection, the GFP signals were observed using a laser scanning confocal microscope (LSM 880, Zeiss, Germany), and the gene-specific clone primers are listed in Supplement Table S2.

## 5. Conclusions

In this study, we identified and analyzed a total of 57 *FvLAC* genes in the *Fragaria vesca* genome, focusing on their physical and chemical properties, phylogenetic relationships, gene structure, conserved motifs, chromosomal localization, gene duplications, and cis-acting elements. Furthermore, we assessed the expression levels from 20 *FvLAC* genes in groups I, II, and III in response to abiotic stress and hormone treatments, revealing that these genes are responsive to salt, drought, ABA, and MeJA stresses. Notably, the expression levels of *FvLAC24*, *FvLAC32*, and *FvLAC51* consistently increased within 12 h following stress exposure. Transgenic yeast experiments demonstrated that *FvLAC51* enhances yeast tolerance to salt and drought, while *FvLAC24* and *FvLAC51* negatively regulate yeast tolerance under these conditions. Subcellular localization experiments indicated that FvLAC24, FvLAC32, and FvLAC51 are situated within the cell membranes. These findings offer significant insights into the evolutionary processes, expansion, regulation, and expression patterns of *FvLAC* genes in the woodland strawberry, which provide valuable information for further investigation into the functions of *FvLAC* genes in the woodland strawberry.

## Figures and Tables

**Table 1:** Molecular characterization of *FvLAC* family genes and the corresponding proteins in *Fragaria vesca*.

Gene Name	Gene ID	Position	Protein Length (aa)	MW (kDa)	pI (Isoelectric Point)	Aliphatic Index	Grand Average of Hydropathicity (GRAVY)	Subcellular Localization
FvLAC1	FvH4_1g13060.t1	Fvb1: 7195820-7198114	566	62.59965	8.81	81.11	−0.142	Cell Membrane
FvLAC2	FvH4_1g14980.t1	Fvb1: 8412006-8415378	537	59.63128	9.49	89.11	−0.084	Cell Membrane
FvLAC3	FvH4_1g14990.t1	Fvb1: 8418523-8421631	539	60.25449	9.47	84.25	−0.253	Cell Membrane
FvLAC4	FvH4_1g20010.t1	Fvb1: 12113062-12129311	568	62.75757	4.92	77.75	−0.182	Cell Membrane
FvLAC5	FvH4_1g21540.t1	Fvb1: 13515806-13518950	588	65.9508	6.78	81.19	−0.235	Cell Membrane
FvLAC6	FvH4_1g24380.t1	Fvb1: 16242663-16247257	574	64.03903	8.02	81.69	−0.224	Cell Membrane
FvLAC7	FvH4_2g08670.t1	Fvb2: 7595223-7598610	562	62.08846	8.91	86.58	−0.037	Cell Membrane
FvLAC8	FvH4_2g08840.t1	Fvb2: 7714665-7717273	563	62.15149	9.07	85.22	−0.075	Cell Membrane
FvLAC9	FvH4_2g12570.t1	Fvb2: 11035380-11040775	526	57.81445	5.41	82.09	−0.128	Cell Membrane
FvLAC10	FvH4_2g12590.t1	Fvb2: 11047104-11051161	565	62.70119	6.18	79.84	−0.144	Cell Membrane
FvLAC11	FvH4_2g12610.t1	Fvb2: 11066186-11071363	567	62.35179	7.04	77.6	−0.123	Cell Membrane
FvLAC12	FvH4_2g12620.t1	Fvb2: 11082038-11085711	593	66.66999	4.71	73.31	−0.199	Cell Membrane
FvLAC13	FvH4_2g12640.t1	Fvb2: 11096982-11100699	597	67.32025	5.09	79.11	−0.156	Cell Membrane
FvLAC14	FvH4_2g12820.t2	Fvb2: 11263752-11267345	586	65.90307	4.78	72.29	−0.282	Cell Membrane
FvLAC15	FvH4_2g12830.t1	Fvb2: 11269702-11275196	615	69.20619	5.04	78.37	−0.22	Cell Membrane
FvLAC16	FvH4_2g16000.t1	Fvb2: 13973659-13977994	540	60.65865	9.29	81.89	−0.296	Cell Membrane
FvLAC17	FvH4_2g17700.t1	Fvb2: 15202774-15207062	588	65.01755	5.45	88.35	−0.049	Cell Membrane
FvLAC18	FvH4_2g18990.t1	Fvb2: 16155342-16159380	518	58.02463	5.13	82.16	−0.141	Cell Membrane
FvLAC19	FvH4_2g21650.t1	Fvb2: 17953843-17956181	586	64.92625	5.22	81.16	−0.21	Cell Membrane
FvLAC20	FvH4_2g37780.t1	Fvb2: 27427351-27432183	545	60.58551	9.2	86.9	−0.219	Cell Membrane
FvLAC21	FvH4_2g40270.t1	Fvb2: 28723626-28726549	616	69.37165	5.37	75.45	−0.186	Cell Membrane
FvLAC22	FvH4_3g05920.t1	Fvb3: 3441792-3444593	581	64.42843	9.33	92.62	−0.018	Cell Membrane
FvLAC23	FvH4_3g14660.t1	Fvb3: 9038326-9041336	579	65.03623	8.01	84.97	−0.19	Cell Membrane
FvLAC24	FvH4_3g34100.t1	Fvb3: 29509155-29511629	527	58.61884	9.34	94.5	−0.038	Cell Membrane
FvLAC25	FvH4_3g34310.t1	Fvb3: 29812823-29815653	581	64.54466	9.36	93.27	−0.015	Cell Membrane
FvLAC26	FvH4_3g34340.t1	Fvb3: 29823780-29826764	584	64.35617	9.33	89.76	−0.044	Cell Membrane
FvLAC27	FvH4_3g38140.t1	Fvb3: 32569772-32573695	561	61.86636	9.05	89.96	−0.044	Cell Membrane
FvLAC28	FvH4_3g39750.t2	Fvb3: 33703382-33706783	578	63.68979	8.72	83.2	−0.106	Cell Membrane
FvLAC29	FvH4_3g46080.t1	Fvb3: 38231205-38234498	552	61.78519	7.67	78.03	−0.369	Cell Membrane
FvLAC30	FvH4_4g22740.t1	Fvb4: 25453431-25457792	593	66.87838	5.33	80	−0.21	Cell Membrane
FvLAC31	FvH4_5g00300.t1	Fvb5: 211106-215572	590	65.88286	8.9	85.73	−0.202	Cell Membrane
FvLAC32	FvH4_5g17150.t1	Fvb5: 9786606-9789346	581	65.54888	8.6	76.82	−0.278	Cell Membrane
FvLAC33	FvH4_5g29070.t2	Fvb5: 20173849-20178790	578	63.94649	7.39	80.09	−0.278	Cell Membrane
FvLAC34	FvH4_5g35760.t2	Fvb5: 26184996-26188385	589	66.24063	5.07	75.59	−0.237	Cell Membrane
FvLAC35	FvH4_5g36460.t1	Fvb5: 26744115-26747456	590	66.75552	5.29	86.32	−0.164	Cell Membrane
FvLAC36	FvH4_5g36541.t1	Fvb5: 26803908-26806116	440	49.19912	4.84	76.86	−0.243	Cell Membrane
FvLAC37	FvH4_6g11960.t1	Fvb6: 7170306-7173452	561	61.07522	9.36	91.39	−0.016	Cell Membrane
FvLAC38	FvH4_6g12410.t1	Fvb6: 7488545-7491071	565	61.93886	6.78	91.45	0.05	Cell Membrane
FvLAC39	FvH4_6g12430.t1	Fvb6: 7501544-7504444	565	61.79521	6.03	88.71	0.002	Cell Membrane
FvLAC40	FvH4_6g13390.t1	Fvb6: 8140343-8143219	591	65.33345	9.78	81.76	−0.143	Cell Membrane
FvLAC41	FvH4_6g13430.t1	Fvb6: 8150815-8155584	585	64.61023	9.33	84.32	−0.118	Cell Membrane
FvLAC42	FvH4_6g20700.t1	Fvb6: 14273906-14275558	550	61.6282	8.34	81.55	−0.319	Cell Membrane
FvLAC43	FvH4_6g20720.t1	Fvb6: 14290925-14292572	466	51.90107	8.95	79.1	−0.315	Cell Membrane
FvLAC44	FvH4_6g35560.t1	Fvb6: 28057302-28057449	460	51.55499	4.52	83.65	−0.175	Cell Membrane
FvLAC45	FvH4_6g39950.t1	Fvb6: 31547912-31551090	592	66.35409	4.64	82.01	−0.076	Cell Membrane
FvLAC46	FvH4_6g39970.t1	Fvb6: 31557548-31561494	594	66.73349	4.95	76.3	−0.178	Cell Membrane
FvLAC47	FvH4_6g39983.t1	Fvb6: 31578149-31580632	595	66.64642	5.01	81.73	−0.109	Cell Membrane
FvLAC48	FvH4_6g39990.t1	Fvb6: 31584585-31587084	600	67.20496	4.8	82.2	−0.134	Cell Membrane
FvLAC49	FvH4_6g42990.t2	Fvb6: 33349236-33353616	542	60.26189	8.37	80.68	−0.238	Cell Membrane
FvLAC50	FvH4_7g07750.t1	Fvb7: 7643077-7645694	575	63.70139	9.06	76.45	−0.285	Cell Membrane
FvLAC51	FvH4_7g07800.t1	Fvb7: 7696581-7699286	574	63.57031	9.05	76.93	−0.282	Cell Membrane
FvLAC52	FvH4_7g16960.t1	Fvb7: 14530357-14533065	574	63.63589	8.23	82.46	−0.087	Cell Membrane
FvLAC53	FvH4_7g18341.t1	Fvb7: 15320904-15323455	564	63.01902	4.88	85.82	−0.13	Cell Membrane
FvLAC54	FvH4_7g18350.t1	Fvb7: 15330255-15333363	604	66.8756	6.05	85.03	−0.169	Cell Membrane
FvLAC55	FvH4_7g23980.t1	Fvb7: 18576510-18579531	560	61.70672	7.3	92.07	−0.025	Cell Membrane
FvLAC56	FvH4_7g25330.t1	Fvb7: 19368353-19371071	574	63.25964	8.23	77.09	−0.254	Cell Membrane
FvLAC57	FvH4_7g27730.t1	Fvb7: 20684142-20687949	594	66.40308	6.86	82.88	−0.204	Cell Membrane

**Table 2:** List of *FvLACs* with putative miRNA target sites in *Fragaria vesca*.

Gene	Gene ID	Predicted miRNA Target Sites	miRNA Length	Expectation	Type of Inhibition
FvLAC1	FvH4_1g13060.t1	miR397a	21	3.5	Cleavage
FvLAC3	FvH4_1g14990.t1	miR857	21	4.5	Cleavage
FvLAC7	FvH4_2g08670.t1	miR397a, miR397b	21	1–2.5	Cleavage
FvLAC7	FvH4_2g08670.t1	miR857	21	4.5	Cleavage
FvLAC8	FvH4_2g08840.t1	miR397a, miR397b	21	2–3.5	Cleavage
FvLAC23	FvH4_3g14660.t1	miR857	21	5	Translation
FvLAC26	FvH4_3g34340.t1	miR397b	21	4	Cleavage
FvLAC27	FvH4_3g38140.t1	miR397a, miR397b	21	2–3.5	Cleavage
FvLAC28	FvH4_3g39750.t2	miR397a, miR397b	21	2.5–4	Cleavage
FvLAC32	FvH4_5g17150.t1	miR397a	21	3.5	Cleavage
FvLAC34	FvH4_5g35760.t2	miR857	21	5	Translation
FvLAC37	FvH4_6g11960.t1	miR397a, miR397b	21	1–2.5	Cleavage
FvLAC38	FvH4_6g12410.t1	miR397a, miR397b	21	0–1.5	Cleavage
FvLAC39	FvH4_6g12430.t1	miR397a, miR397b	21	1.5–3	Cleavage
FvLAC41	FvH4_6g13430.t1	miR397a, miR397b	21	1.5–3	Cleavage
FvLAC41	FvH4_6g13430.t1	miR857	21	4.5	Cleavage
FvLAC52	FvH4_7g16960.t1	miR857	21	5	Cleavage
FvLAC55	FvH4_7g23980.t1	miR397a	21	4	Cleavage

## Data Availability

All the accession numbers of the woodland strawberry (*Fragaria vesca*) *FvLAC* genes in this study can be found in Table 1.

## Supplementary Materials

The following supporting information can be downloaded at: https://www.mdpi.com/article/10.3390/plants13233366/s1, Table S1: Cis-elements analysis of *FvLAC* gene promoters; Table S2: The primers used in this study.
